# Predictors of drug prescription in nursing home residents: results from the INCUR study

**DOI:** 10.1007/s11739-021-02841-6

**Published:** 2021-09-21

**Authors:** Laura Calcaterra, Marco Proietti, Edoardo Saporiti, Vanessa Nunziata, Yves Rolland, Bruno Vellas, Matteo Cesari

**Affiliations:** 1 Geriatric Specialty Training School, University of Milan, Milan, Italy; 2 Geriatric Unit, Fondazione IRCCS Istituti Clinici Scientifici Maugeri, Via Camaldoli 64, 20138 Milan, Italy; 3 Department of Clinical Sciences and Community Health, University of Milan, Milan, Italy; 4 Liverpool Centre for Cardiovascular Science, University of Liverpool, Liverpool Heart and Chest Hospital, Liverpool, UK; 5 Geriatria, Accettazione geriatrica e Centro di ricerca per l’invecchiamento, IRCCS INRCA, Ancona, Italy; 6 Gérontopôle de Toulouse, Institut du Vieillissement, Centre Hospitalo-Universitaire de Toulouse, Toulouse, France; 7 UMR INSERM, Toulouse, France

**Keywords:** Older persons, Drugs, Polypharmacy, Frailty, Nursing homes

## Abstract

Polypharmacy represents a major clinical and public health issue in older persons. We aimed to measure the prevalence of polypharmacy, and the main predictors of drug prescription in nursing home residents. Post hoc analyses of the “Incidence of pNeumonia and related ConseqUences in nursing home Residents” (INCUR) study were conducted. Polypharmacy was defined as the prescription of 5 or more drugs. A frailty index (FI) was computed according to the model proposed by Rockwood and Mitnitski using 36 health deficits, including diseases, signs, symptoms, and disabilities. Linear regression models were performed to identify the main predictors of the number of prescribed drugs. The INCUR study enrolled 800 patients (mean [SD] age 86.2 [4.1] years, 74.1% women). The mean number of medications prescribed at the baseline was 8.5 (SD 4.1). Prevalence of polypharmacy was found 86.4%. The mean FI was 0.38 (SD 0.10). A fully adjusted linear multivariate regression model found an inverse and independent association between age and number of prescribed drugs (beta − 0.07, 95% CI − 0.13, − 0.02; *p* = 0.005). Conversely, the FI was independently and positively associated with the number of medications (beta 4.73, 95% CI 1.17, 8.29; *p* = 0.009). The prevalence of polypharmacy is high among older persons living in nursing home. Age and FI are significantly associated with the number of drugs. The number of prescribed drugs tends to decrease with age, whereas a direct association with frailty is reported.

## Introduction

Polypharmacy is the concomitant use of multiple drugs by a single individual and represents a highly prevalent condition in the older population. A systematic review by Jokanovic et al. reported that the prevalence of polypharmacy ranges from 38 to 91% in institutionalized individuals [1]. Although there are many definitions of polypharmacy, it is commonly defined as the use of five or more drugs [2].

Numerous studies have shown that polypharmacy is associated with adverse outcomes, including mortality, falls, and prolonged hospital stay [3]. Among the possible mechanisms provided to explain this, the age-related changes in pharmacokinetics and pharmacodynamics increasing the risk of adverse drug reactions, are considered among the most relevant causes. Different factors have been associated with polypharmacy. In particular, multimorbidity and increased clinical complexity tend to increase the use of drugs, whereas age and disability reduce drug consumption [1].

Frailty is defined as a state of increased vulnerability to stressors as a result of a decrease in physiologic reserves [4]. There are several models used to define and measure frailty. Among the different models, the frailty index (FI), proposed by Rockwood et al. [5], is one of the most commonly used. The FI is based on the rationale that the more deficits a person accumulates with aging, the more likely that person will be frail (or biologically old). A bidirectional association between frailty and polypharmacy in older adults has been demonstrated [6]. Nursing home residents constitute an extremely vulnerable population, characterized by multiple chronic diseases, thus likely to be exposed to polypharmacy (and its clinical consequences) [7].

The aim of this study was to determine the prevalence of polypharmacy in nursing home resident and identify the main predictors of drug prescription in this specific population.

## Methods

The data used in this study were collected as part of the INCUR study, a multicentre observational cohort study. The study rationale and design have been previously described [8]. Briefly, the aim of the INCUR study was to estimate the incidence of pneumonia events in older persons living in nursing homes over a period of 12 months. A total of 800 nursing home residents aged 60 and older were recruited in 13 nursing homes randomly selected in the Midi-Pyrenees region of France between 2012 and 2013. The data that were collected included sociodemographic and lifestyle characteristics, chronic diseases, and functional status. The Ethics Committee of the Toulouse University Hospital and the Consultative Committee for the Treatment of Research Information on Health (CNIL) approved the entire study protocol. No formal written informed consent was needed, as the data collected were part of daily standard care activities; nonetheless, all participants were informed about the ongoing research and could eventually withdraw from the participation.

At the baseline assessment, the study personnel recorded all the drugs included in the participants’ therapeutic plan, referring to the regularly administered drugs. All the drugs were coded using the Anatomical Therapeutic Chemical (ATC) code system. In this study, polypharmacy was defined by the presence of 5 or more drugs. We also assessed polypharmacy according to other reported cut-offs [9].

Results from the comprehensive geriatric assessment performed at the baseline visit were also available and included the Abbreviated Mental Test Score (AMTS), the Geriatric Depression scale (GDS), the Activities of Daily Living (ADL) scale, and a modified version of the Instrumental ADL (IADL) scale.

A 36-item FI was computed according to the model proposed by Mitnitski and Rockwood [5], standardized according to the criteria described by Searle et al. [10]. Details about the items considered for frailty index see Table 1.

**Table 1:** Items included into the frailty index

Variable	Definition	Deficit value
Hypertension	Present	1
Atrial fibrillation	Present	1
Coronary artery disease	Present	1
History of myocardial infarction	Present	1
Heart failure	Present	1
Peripheral neuropathy	Present	1
Depression	Present	1
Arthritis	Present	1
Osteoporosis	Present	1
Chronic respiratory failure	Present	1
Chronic obstructive pulmonary disease	Present	1
Chronic kidney disease	Present	1
Dementia	Present	1
Parkinson’s disease	Present	1
Any Thyroid disease	Present	1
Diabetes mellitus	Present	1
Liver disease	Present	1
Any neoplasm	Present	1
History of cerebrovascular events	Present	1
Vision impairment	Present	1
Hearing impairment	Present	1
Activities of daily living	6–0	0–6
Instrumental activities of daily living	4–0	0–4
SPPB gait speed	Unable to Perform	1
SPPB balance	Unable to Perform	1
SPPB chair stand	Unable to Perform	1
Unintended weight loss	Present	1
Abbreviated mental test score	≤ 7	1

*SPPB* Short Physical Performance Battery

### Statistical analysis

Differences in continuous variables were evaluated according to the ANOVA test, while differences in categorical variables were tested according to Chi-square test. Correlation analysis was performed according to Pearson’s model.

Linear regression models were performed to explore the factors associated to the number of drugs prescribed. Age, sex, and education were considered as potential confounders given their relationship with an increased use of medications [11]. A two-sided *p* value < 0.05 was considered as statistically significant. Statistical analyses were performed using SPSS v24 software.

## Results

The INCUR study enrolled a total of 800 nursing home residents. The mean age of the study sample was 86.2 years (SD 4.1), with a large predominance of women (74.1%. The main characteristics of the study sample are shown in Table 2.

**Table 2:** Baseline characteristics of patients included in the study

Variable	*N* = 800
Age, years mean (SD)	86.2 (4.1)
Female sex, *n* (%)	593 (74.1)
ADL, mean (SD)	3.6 (1.8)
IADL, mean (SD)	3.4 (0.7)
Frailty index, mean (SD)	0.38 (0.10)
Drugs, *n* mean (SD)	8.48 (4.10)
Pain score, mean (SD)	59 (33.7)
GDS, mean (SD) *582*	2.9 (2.4)
AMTS, mean (SD) *718*	5.6 (3.6)

Italic numbers are referred to the non-missing values for each variable

*ADL* Activities of Daily Living, *AMTS* Abbreviated Mental Test Score, *GDS* Geriatric Depression Scale, *IADL* Instrumental Activities of Daily Living, *SD* standard deviation

**Fig. 1:**
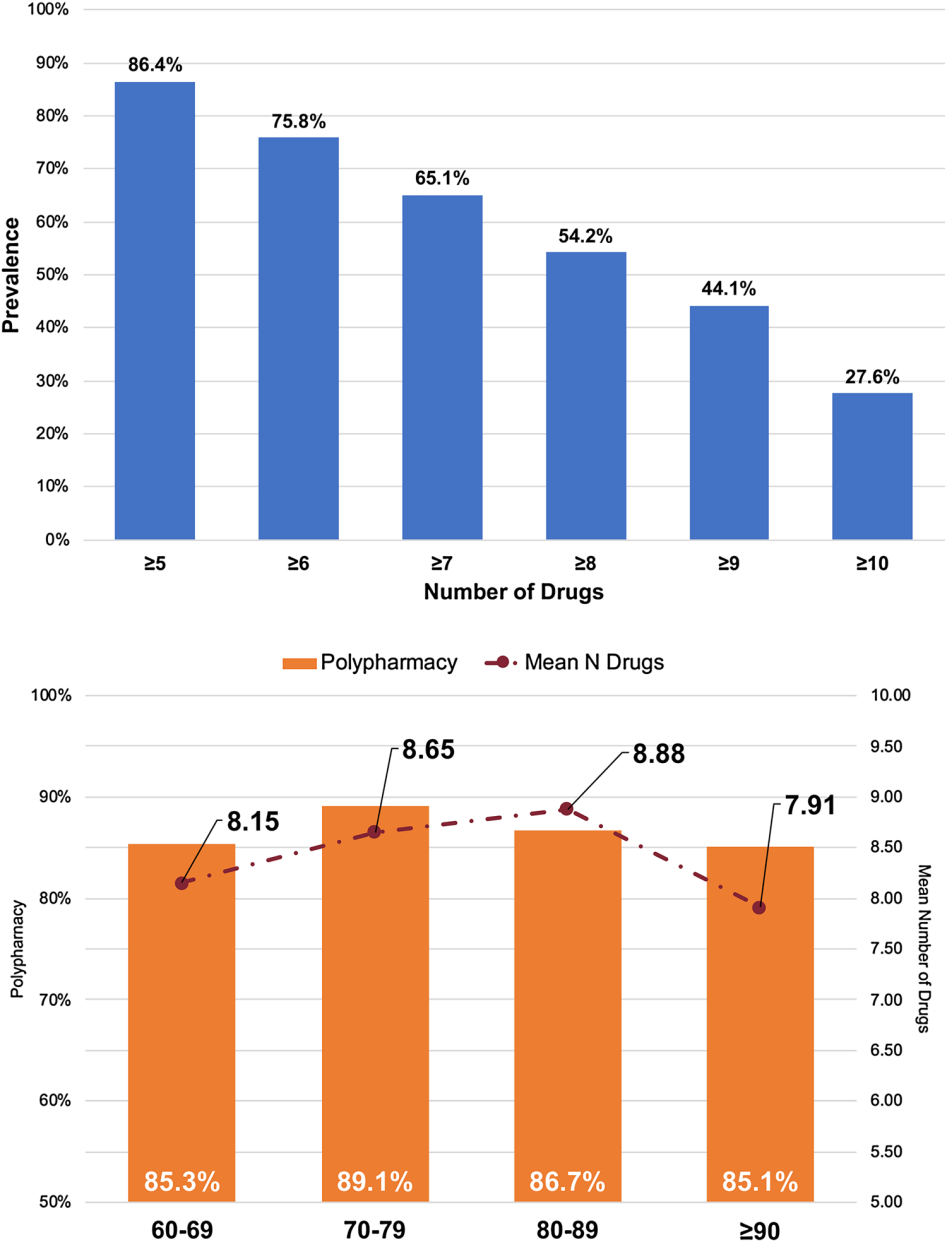
Prevalence of polypharmacy and number of drugs according to age classes

A high prevalence of polypharmacy was present (86.4%). The mean number of medications prescribed at baseline was 8.5 (SD 4.1, range 1–25). Using progressively higher cut-off for polypharmacy definition, we reported a progressively lower prevalence, up to 27.6% when the concomitant use of 10 or more drugs was considered [Fig. 1, Upper Panel].

Looking at the prevalence of polypharmacy (≥ 5 drugs) across age groups, no statistical difference was reported (*p* = 0.792) [Fig. 1, Lower Panel]. At the same time, the mean number of drugs tended to decrease with aging, and the oldest residents (i.e., 90 years or older) took a significantly lower number of drugs (*p* = 0.02) [Fig. 1, Lower Panel].

**Fig. 2:**
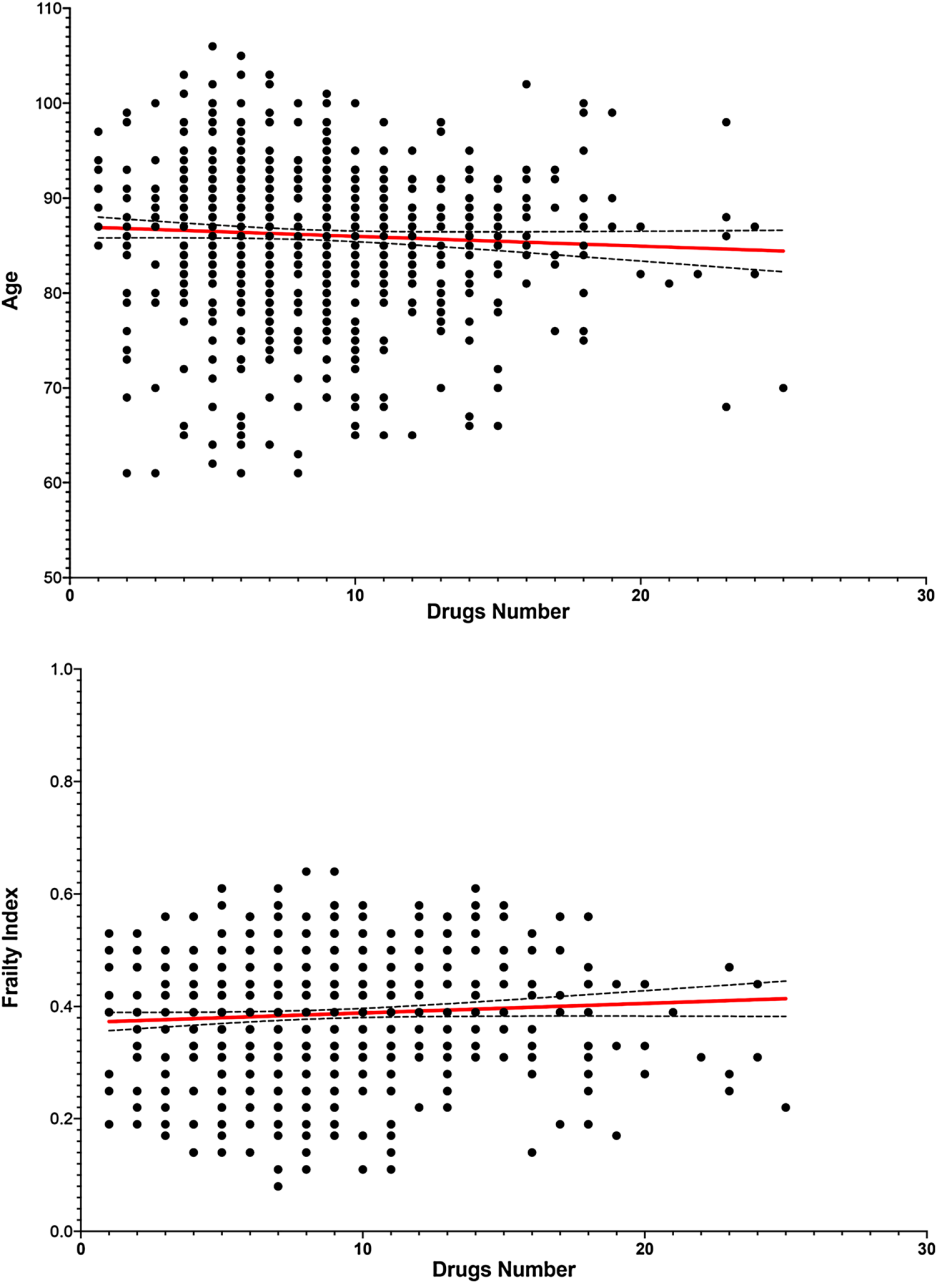
Scatterplot and correlation analysis between age, frailty index and number of drugs

Pearson’s models showed a non-significant, inverse correlation between age and the number of drugs (*r* = − 0.056, *p* = 0.12) [Fig. 2, Upper Panel]. A non-significant direct association between FI (*r* = 0.067, *p* = 0.08) and the number of drugs was also reported [Fig. 2, Lower Panel].

A fully adjusted linear regression model, including age, sex, education years and FI as possible predictors of the number of prescribed medications, showed an inverse and independent association between age and the number of drugs (Table 3). At the same time, the FI was independently and positively associated with the number of prescribed drugs (Table 3).

**Table 3:** Multivariate linear regression analysis

	Number of concomitant medications			
	Beta	95% CI	*T*	*p*
Age	− 0.07	− 0.13/− 0.02	− 2.782	0.005
Female sex	0.18	− 0.65/1.01	0.417	0.677
Education years	0.02	− 0.09/0.14	0.393	0.694
Frailty index	4.73	1.17/8.29	2.508	0.012

*CI* Confidence Interval

## Discussion

In this cohort of nursing home residents, we found a high prevalence of polypharmacy with almost 9 out of 10 patients being prescribed with 5 or more drugs. Among the possible predictors, age was inversely associated with the number of drugs prescribed, whereas FI showed a direct association. To our knowledge, this study represents the first evaluation of the relationship between the concept of frailty, as age-related accumulation of deficits, and drugs prescription in European nursing home residents.

In a systematic review investigating the prevalence and associated factors of polypharmacy in older subject in long-term care facilities [1], while underlining the large heterogeneity in definitions used among the 44 studies included, it was showed how the prevalence in this setting is widely ranging [1]. In our cohort, the prevalence of polypharmacy is high and remains high even when different defining criteria are applied.

Our study shows that increasing age is associated with a reduced number of prescribed medications, in particular in residents aged 90 years or older. Our results are consistent with the above-mentioned review by Jokanovic et al. showing the inverse relationship between age and drugs prescription [1]. This finding is also similar with the results from the ‘Services and Health for Elderly in Long TERm care’ (SHELTER) [7]. This result can be explained by the increasing awareness about the need of discontinuing unnecessary drugs in older and oldest old patients [12], as well as the lack of data on benefits of many treatments in frail older individuals. In fact, it is known how the frail and/or oldest old persons are poorly (if not) represented in large pharmacological trials, leaving large areas of uncertainty [13]. Furthermore, drug prescription may be guided by the limited life expectancy of these specific patients [14].

In this study, frailty was independently and positively associated with the number of medications. The more the patient was frail, the higher was his/her likelihood of being prescribed with more drugs. Similar evidence has already been reported in other studies [7, 15, 16]. A large meta-analysis demonstrated a bidirectional relationship between polypharmacy and frailty [6, 16]. Notwithstanding, the largest proportion of studies included in the analysis were primarily exploring the phenotypic model of frailty proposed by Fried et al. [17]. Another review which further analyzed this relationship showed again that, in this context, frailty is almost always defined as a discrete variable [18]. In our study, frailty was framed according to the Mitnitski and Rockwood model [5], that synthetizes the concepts of biological age and clinical complexity [5]. Our study shows that the more the patient is clinically complex, the more drugs him/her receives. So far, the use of FI to measure frailty in this specific area has been scarcely applied and exclusively in East Asian community-dwelling populations [19, 20]. Thus, this paper confirms and extends previous knowledge, confirming that clinical complexity drives pharmacological management, even in nursing home residents, as opposed to previous studies that did not show such an association in this specific clinical context [21].

We can postulate that this phenomenon is determined by the obsolete single disease approach, which aims to treat the patients for each condition they have according to specific specialist guidelines, rather than adopting an integrated and holistic approach, targeting the overall health state of the oldest old people [22]. Furthermore, it is paradoxical the decrease of prescriptions with increasing age, but the higher number of prescribed drugs among the frailest individuals. This might potentially indicate that clinicians are still driven in their deprescribing by the concept of chronological age of the subject but tend to remain hesitant at reducing the number of medications in front of the clinical complexity (despite the evident evidence-based medicine issue in nursing home).

The relationship between frailty and polypharmacy is bidirectional. First, the accumulation of comorbidities and chronic diseases can lead to frailty and over-prescription for every single disease. Second, polypharmacy may contribute to frailty as it has been linked to hospitalization, low therapeutic adherence and adverse drug reactions (ADR), all known causes of increased frailty [6]. ADR lead to a prescribing cascade in which new medications are prescribed to counteract unexpected effects of the initial drug, possibly leading to frailty [23].

We believe that our paper has important potential clinical implications. Indeed, the concomitant use of more medications is associated with an increased risk of drug-to-drug interactions [24] and even though were defined as “uncommon”, the drug-to-drug-interactions have been reported as a relevant clinical issue in clinically complex patients [25], causing potentially serious adverse events. Such evidence is also confirmed by other studies which also documented how potentially inappropriate medications according to Beers’ criteria are much more common in frail nursing homes residents [26]. The data of such high prevalence of polypharmacy in out cohort poses the need for more medication revision and reconciliation.

There are several limitations to this study. First, our population was recruited in one region of France; our results might thus not be directly applicable elsewhere. Second, the cross-sectional nature of our study does not allow to speculate on the cause–effect direction of the investigated relationship. Lastly, the subjects included had a very high age, hence the evidence about the inverse relationship between age and prescribed drugs may not be fully generalizable. Further research is needed to confirm the possible benefits of reducing polypharmacy in the development, reversion or delay of frailty.

In conclusion, age does not negatively contribute to the problem of polypharmacy, whereas frailty (i.e., clinical complexity) is associated to an increased number of drugs prescribed. Frail persons are at risk of receiving too many prescriptions, increasing the risk of negative health-related outcomes. Among nursing home residents, regular evaluation of frailty, as well as the implementation of a deprescribing process, could help reducing the burden of adverse outcomes associated with polypharmacy.

## Data Availability

All relevant data have been published in the paper.
